# Pathogenesis of multimorbidity—what is known?

**DOI:** 10.1007/s00391-020-01752-z

**Published:** 2020-07-10

**Authors:** Tilman Wetterling

**Affiliations:** 1 Vivantes Klinikum Kaulsdorf, Berlin, Germany; 2 ZIP-Klinik für Psychiatrie und Psychotherapie UKSH, Lübeck, Germany

**Keywords:** Multimorbidity, Risk factors, Harmful behaviors, Model of concepts, Therapeutical implications, Multimorbidität, Risikofaktoren, Schädliche Verhaltensweisen, Konzeptmodell, Therapeutische Möglichkeiten

## Abstract

**Background:**

Multimorbidity is gaining increasing attention due to its substantial medical, healthcare political and social challenges. So far, however, there have been only few studies attempting to characterize the underlying pathogenesis.

**Method:**

A selective literature search was carried out in PubMed.

**Results:**

There is no generally accepted definition of multimorbidity. In the studies published so far, attempts have mostly been made to identify frequent clusters of diseases. In order to prevent multimorbidity, however, it is necessary to characterize the underlying mechanisms of development in more detail. For this purpose, a concept is presented based on the previously published data, in particular from longitudinal studies showing the importance of known risk factors. Possible pathogenetic processes involving multimorbidity are briefly discussed.

**Conclusion:**

For most pathogenetic processes leading to multimorbidity there is no suitable pharmacological treatment available; however, behavior such as lack of exercise, dietary habits, smoking and high alcohol consumption are of considerable importance for the development of multimorbidity and can in principle be influenced by treatment.

## Abstract (de)

**Hintergrund:**

Das Thema Multimorbidität gewinnt aufgrund der großen damit verbundenen medizinischen, gesundheits- und sozialpolitischen Herausforderungen zunehmend an Aufmerksamkeit. Bisher gibt es aber nur wenige Studien, in denen versucht wurde, die Entstehungsmechanismen näher zu charakterisieren.

**Methode:**

Selektive Literaturrecherche in PubMed und einige Beispielberechnungen auf der Basis eigener Daten (GePsy-Studie).

**Ergebnisse:**

Noch gibt es keine allgemein anerkannte Definition für Multimorbidität. In den bisher vorliegenden Untersuchungen wurde vor allem versucht, häufige Kombinationen von Erkrankungen (sog. Cluster) zu identifizieren. Zur Prävention und Behandlung der Multimorbidität ist es aber notwendig, die vielfältigen Entstehungsmechanismen näher zu charakterisieren, um so eine Differenzierung vornehmen und entsprechende therapeutische Strategien entwickeln zu können. Hierzu wird ein Konzept vorgestellt, das auf den bisher publizierten Daten, insbesondere aus Verlaufsstudien beruht. Diese zeigen die Bedeutung bekannter Risikofaktoren. Mögliche, einer Multimorbidität zugrundeliegende pathogenetische Prozesse werden kurz dargestellt.

**Schlussfolgerungen:**

Die vielfältigen pathogenetischen Prozesse, die zu einer Multimorbidität führen können, sind bisher medikamentös kaum beeinflussbar. Aber Verhaltensweisen wie Bewegungsmangel, Ernährungsgewohnheiten, Rauchen und hoher Alkoholkonsum sind für die Entwicklung einer Multimorbidität von erheblicher Bedeutung und prinzipiell therapeutisch beeinflussbar.

For affected persons multimorbidity is a major burden that leads to impairment of the quality of life, including mobility and restrictions on self-sufficiency. As a result, mental disorders, particularly depression, often occur [4]. Multimorbidity is also commonly associated with cognitive disorders [12] and leads to increased mortality [29]. In view of these facts, the number of scientific publications on the topic of multimorbidity has significantly increased in recent years.

A difficulty in considering the studies is that a generally accepted definition of multimorbidity and standardized assessment tools has so far been lacking. This is due to a variety of aspects to be taken into account. In most proposals for a definition (see overview [24, 28]), multimorbidity is defined as the simultaneous presence of three or more chronic diseases [6]. A closer look at the different definitions reveals that a wide range of physical impairments are also included [28]: risk factors such as hyperlipidemia and symptoms such as back pain. In Anglo-American literature, therefore, the term multiple chronic conditions is used as well. It is also not clearly defined what is meant by chronic. In most cases, a duration of at least 1 year is considered to be necessary.

Most of the previous studies on multimorbidity are cross-sectional ones related to the actual state, the conditions of occurrence (e.g. age, education, socioeconomic status) and the consequences of multimorbidity, in particular increased frailty and mortality during the course [30]. In order to design strategies for prevention and treatment, probable routes of development of multimorbidity and possible underlying pathomechanisms have be elucidated.

## Methods

**Fig. 1:**
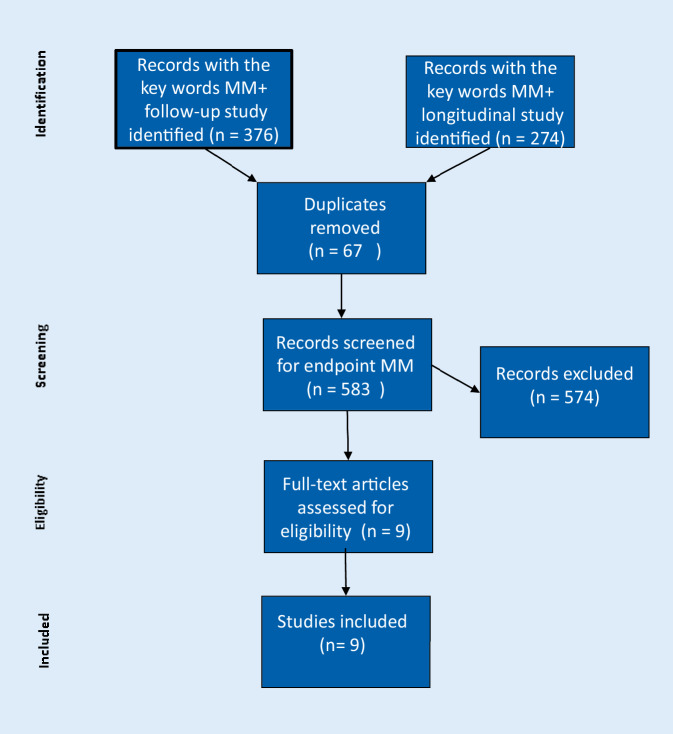
PRISMA flow diagram showing the search strategy. *MM* multimorbidity, *MM+* second keyword

A systematic review was carried out by searching PubMed for articles with the key words multimorbidity, longitudinal and follow-up study (Fig. 1). The retrieval of relevant studies was conducted via title screening, abstract screening, and full-text eligibility assessment applying the defined inclusion criterion endpoint multimorbidity. The literature research (conducted on 15 August 2019) yielded 583 publications.

## Results

Clues for the development of multimorbidity can be obtained by two different ways:

### 1) Investigations in cross-sectional studies showing statistical relationships of the different diseases in multimorbidity

The associations of diseases in multimorbidity are very complex and can only be calculated using different mathematical models (e.g. cluster or factor analyses). The clusters of the most common combinations of diseases show a considerable heterogeneity [23]. A recent review of 51 studies identified a total of 407 different multimorbidity profiles [5].

**Fig. 2:**
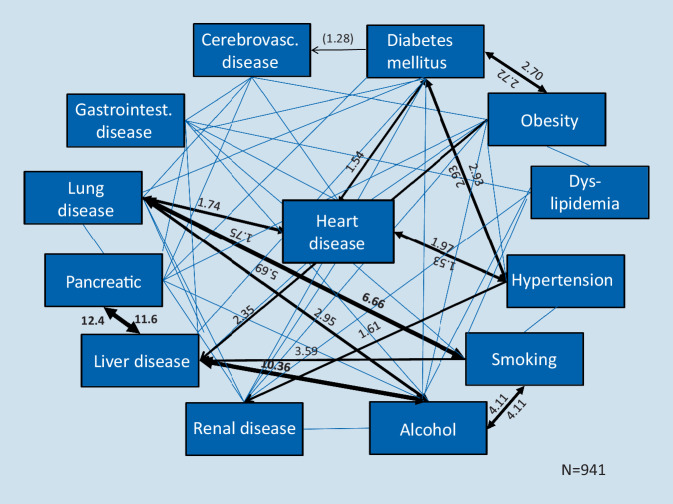
Relations between risk factors and diseases of different organs. Diagramed are all relations with odds ratios >1 (*blue lines*), in case of significant correlation with the odds ratio (*black lines*)

Attempts were made to visualize the relationships between the diseases using various methods for calculation, i.e. the strength of the statistical relationship of corresponding diseases [19]. For illustration see Fig. 2, calculated on previously published own data [25]. Due to the complexity, i.e. the variety of combination options, a tabular form is also suitable for an overview. Additional data, such as age group and statistical correlations (odds ratios) can increase the significance [3].

These descriptions of the actual state, however, say little about the development of multimorbidity. Above all, they show the manifold interrelationships. These can be used, especially when respecting different age groups, for initial considerations and hypotheses on etiology. For this purpose, correlations with high odds ratios have to be primarily evaluated; however, meaningful data on the etiology of multimorbidity can only be obtained from longitudinal studies. Our data (shown in Fig. 2) revealed more significant relationships between risk factors and diseases than interrelationships of different organs diseases.

### 2) Follow-up studies with the endpoint multimorbidity

An Australian study [16] attempted to clarify which disease was the first to occur in later manifest multimorbidity. The results showed a heterogeneous picture with hyperlipidemia as the most common first manifestation. Other longitudinal studies (Table 1) mainly considered behavioral disorders, i.e. behavior that differs from the normal population or from the recommendations of medical societies (lack of physical activity, smoking, increased alcohol consumption and obesity or dietary habits). These increase the likelihood of multimorbidity developing during follow-up.

**Table 1:** Longitudinal studies (endpoint multimorbidity)

Author	Study population	Developing multimorbidity	Age at baseline (years)	Follow-up (years)	Risk factors	Hazard ratio (adjusted) (confidence interval)	
Dhalwani et al. [7]	England 5476	1156 (21.1%)	>50	Ø3.5	Physical inactivity + overweight	2.87 (95% CI 1.55–5.31)	
					Physical inactivity + smoking	2.35 (95% CI 1.35–4.08)	
					Physical inactivity + overweight + smoking	3.98 (95% CI 1.02–17.00)	
Katikireddi et al. [10]	Scotland 2604	–	35	20	Smoking	1.57 (95% CI 1.37–1.80)	
					Alcohol (>21 units/week)	1.49 (95% CI 1.26–1.76)	
					No fruits or vegetables	1.45 (95% CI 1.24–1.71)	
					Overweight (BMI 25–29.9)	1.26 (95% CI 1.12–1.41)	
					Obesity grade I (BMI 30–34.9)	1.43 (95% CI 1.21–1.68)	
					Obesity grade II/III (BMI ≥35)	1.98 (95% CI 1.50–2.62)	
Kivimäki et al. [11]^a^	16 cohort studies from the USA and Europe 120.813 (59.1% w)	1627 (1.3%)	Ø51.4 (35–103)	Ø10.7	Overweight (BMI 25–29.9)	2.0 (95% CI 1.7–2.4)	
					Obesity grade I (BMI 30–34.9)	4.5 (95% CI 3.5–5.8)	
					Obesity grade II/III (BMI ≥35)	14.5 (95% CI 10.1–21.0)	
Mounce et al. [14]	England 1477	901 (61.0%)	50+	Ø10	Age	No data	
					Physical inactivity	Only trend (*p* = 0.031)	
					Social status (poor vs. wealthy)	2.19 (95% CI 1.50–3.19)	
					Obesity	1.92 (95% CI 1.43–2.59)	
Ryan et al.^c^ [17]	Ireland 2235	–	–	–	Age 60–69	1.30 (95% CI 1.11–1.52)	
					Overweight	1.26 (95% CI 1.05–1.51)	
					Reduction of grip	0.98 (95% CI 0.97–0.99)	
					Reduction of gait velocity	0.67 (95% CI 0.49–0.90)	
Singh-Manoux et al.^a,b^ [18]	England 8270	511 (6.2%)	50+	Ø23.7	Education (no academic qualification) Low occupational position Poor diet Smoking Overweight/obesity Alcohol abuse Hypercholesterolemia	^c^	
Tomasdottir et al. [22]	Norway 20.365	6277 (30.8%)	Ø40.6	11	Financial worries	1.58	
					Low life satisfaction	1.60	
					Not feeling calm and good	1.71	
					Poor self-rated health	2.34	
Wikström et al. [27]	Finland 32.972	–	25–64	10	–	Men	Women
					Current smoker	2.68	2.55
					Physical inactivity (low vs. high)	1.34	1.62
					High BMI	1.11	1.08
					Blood pressure	1.14	1.03^d^
					Education	1.40	1.37^d^
Xu et al.^b^ [30]	Australia 11.914 (only women)	423 (3.6%)	Ø47	20	Marital status	1.55 (95% CI 1.21–1.98)	
					Education (low vs. high)	1.45 (95% CI 1.01–2.10)	
					Obesity (BMI ≥30)	3.01 (95% CI 2.21–4.08)	
					Hypertension	2.19 (95% CI 1.74–2.75)	
					Smoking	1.78 (95% CI 1.31–2.42)	
					Physical inactivity	1.38 (95% CI 1.08–1.86)	
					Cancer	1.49 (95% CI 1.11–1.99)	
					Depression	1.46 (95% CI 1.17–1.83)	
					Arthritis	1.45 (95% CI 1.13–1.86)	
					Asthma	1.34 (95% CI 1.04–1.72)	

*BMI* body mass index, *w* women

^a^Endpoint was cardiovascular multimorbidity. The risk of developing multimorbidity was elevated in cases also suffering from diabetes mellitus

^b^No aOR (adjusted odds ratio) were given, in this table the factors showing differences between no and at least 2 chronic diseases were compiled

^c^Different endpoints were used (healthy to first disease and first disease to cardiometabolic multimorbidity).

^d^Not significant

The longitudinal studies revealed a significant increase in the likelihood of developing multimorbidity with cumulative number of risk factors or, if a chronic disease already exists [7, 9].

### Proposal for a concept model

In view of the complexity of the development of multimorbidity, it seems appropriate to propose a classification into few categories, which consider etiological aspects as far as known. Furthermore, such a classification should facilitate the decision of drug treatment in problematic constellations as well as approaches to prevention. So far, there are only few publications attempting to classify different types of multimorbidity taking into account different etiologies or pathomechanisms [13, 24].

**Fig. 3:**
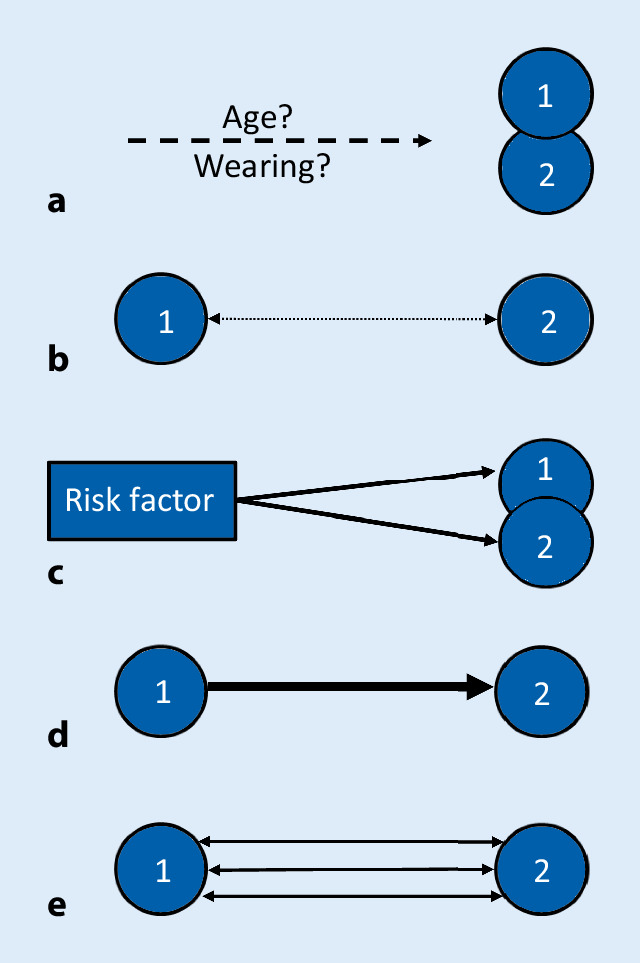
Schema of the different types of multimorbidity (MM) as described in the text. Adapted from [24], modified. **a** *MM type* *1* no common pathogenesis known, **b** *MM type* *2* statistical correlation only, **c** *MM type* *3* known risk factor, **d** *MM type* *4* known causal relation, **e** *MM type* *5* multiple associations. *1* disease 1, *2* disease 2, wearing i.e. deterioration, mechanical stress or fatigue of functionality

The first step towards a concept for categorizing multimorbidity is the comparison of the expected and observed frequency of simultaneous occurrence of diseases on the basis of epidemiological data. If in epidemiological studies the observed frequency of a common occurrence is significantly higher than the expected one, an analysis of possible underlying pathogenesis should be conducted in a second step (see Fig. 3). In order to clarify possible etiological connections, it is helpful to observe the occurrence of the various diseases over time. There are three possibilities basically conceivable: occurrence sequentially, simultaneously and not known.

If there is a combination of two diseases in which, on the basis of epidemiological data, the expected and observed frequencies of simultaneous occurrence do not significantly differ, the probability of mutual influences has to be judged as absent. Thus, the first type of multimorbidity (MM type 0) includes combinations of coexistent impairments of various organs, in which no etiological connection is known.

Often the search for possible common etiological factors reveals only an age dependence of both diseases, so that age may be the essential factor for coexistence. This MM type 1 is often based on physical processes in the broadest sense, e.g. mechanical stress (e.g. osteoarthritis) or fatigue of functionality (e.g. presbycusis, presbyopia).

Moreover, besides age there may be a possibly not yet known association between at least two diseases (MM type 2), but a strong statistical correlation. This type includes a great number of combinations of diseases identified in various cross-sectional studies (see [5, 23]).

The longitudinal studies compiled in Table 1 show the importance of risk factors for the development of multimorbidity. These may become manifested for decades before corresponding diseases occur, e.g. increased alcohol consumption [26]. This type is referred to in the concept presented here as MM type 3. Several different organs may be affected by one single risk factor, e.g. alcohol, smoking or diabetes mellitus. But several risk factors may be responsible for the damage of one organ at the same time as well [22], e.g. in metabolic syndrome.

A disease can also be the result of another, i.e. a kind of causal relation exists, such as in thromboembolic brain infarction in atrial fibrillation. Thus, there is an initial disease (= MM type 4). The organs concerned are often those that are functionally closely related (= systemic multimorbidity). This circumstance often leads to a cascading development of multimorbidity. For example, the disease of one organ (e.g. chronic obstructive pulmonary disease) constantly overwhelms the reserve capacity of another (e.g. heart). Therefore, treatment of the index or first-time disease is particularly important. But here, too, the limitations made above must be taken into account when planning treatment.

A fundamental difficulty in trying to clarify the development of multimorbidity is that connections between the individual diseases are often associative. Frequently, a variety of mostly two-way links between diseases (MM type 5), which can affect each other, are to be taken into account. Often such functional interactions exist not only between two organs, but between several (see Fig. 3). The manifold interactions make adequate treatment planning extremely difficult, especially if a further acute disease (e.g. infection) occurs.

#### Possible pathogenesis

Such a schematic classification of multimorbidity is plausible only if the corresponding pathogenesis is known. A lot of studies show that the prevalence and the incidence of multimorbidity significantly increase with age. Aging is a multifaceted process, involving numerous molecular and cellular mechanisms in the context of different organ systems. Therefore, it seems worthwhile to look at the possible underlying pathogenetic mechanisms of multimorbidity, considering the age dependency:

1. Degenerative intracellular processes (such as shortening of telomeres, mitochondrial dysfunction, etc. [2]. These are especially found in multimorbidity types (1), 2 and 4. Increasing age has the main impact on degenerative processes.
2. Oxidative stress (formation of free radicals, etc.). This is a biochemical process known in some risk factors. It plays an important role, especially in multimorbidity types 2 and 4 [2].
3. Inflammatory processes. These are found in many chronic diseases (e.g. atherosclerosis) [8], which can lead to multimorbidity, especially in types 2 and 4. Functional and structural alterations in the immune system are an important component of aging, i.e. low-grade inflammation.
4. Allergic mechanisms, especially in multimorbidity typse 2 and 4. Aging is associated with a higher prevalence of autoimmunity.
5. Epigenetic mechanisms (e.g. DNA methylation). Recent research has revealed that epigenetic modifications play an important role in the development of physical consequences of risk factors, e.g. diabetes mellitus [1]. Epigenetic mechanisms are particularly important in multimorbidity types 2 and 4.

So far, such studies usually only relate to one disease or a risk factor, but similar mechanisms are described in many studies. Therefore, it is very likely that these pathomechanisms play an important role in the development of multimorbidity. It should be borne in mind that the processes mentioned in 1–5 are closely related or interrelated.

#### Therapeutic implications

So far, there are no drugs sufficiently affecting the majority of the pathobiochemical mechanisms mentioned above. Especially age is an untreatable factor. Therefore, the question arises of currently available options for therapy or prevention of multimorbidity.

Prevention is possible by treating the metabolic risk factors like diabetes mellitus. The guidelines of the corresponding German or European medical societies [15, 20, 21] contain treatment recommendations for cases in which several risk factors are present simultaneously; however, the total number of prescribed drugs, in particular their interactions, as well as age and the limited ability to metabolize must be taken into account.

The available follow-up studies with the endpoint multimorbidity (Table 1) have shown the considerable importance of behavior (such as lack of exercise, smoking) which can ultimately be described as harmful to health. These are not only known risk factors, but also have a negative impact on other risk factors that belong to metabolic syndrome (Table 2). As these risk factors are closely linked, early intervention is required.

**Table 2:** Unhealthy behaviors and (metabolic) risk factors

	Potential interactions			
	Physical inactivity	Smoking	Obesity	Risky alcohol consumption
Prevalence in Germany in people aged 18–64 years	About 30 million	About 15 million	(BMI ≥30) about 10 million	About 3.4 million
*Risk factors*				
Hypertension	++	+	++	++
Diabetes mellitus type II	++	+	+++	++
Hypercholesterolemia	+	–	(+)	–
Hypertriglyceridemia	+	–	+++	++

*BMI* body mass index

Source [24] modified

## Discussion

Until now, little has been published about the complex ways of development of multimorbidity [13, 24]. This is due, among other things, to the large number of socioeconomic, psychosocial and genetic factors as well as age and environment, to be taken into account. Affected persons and the doctors treating them can have little direct influence on these factors and thus contribute to prevention.

In view to developing a concept for the different routes of multimorbidity formation this article highlighted the medical aspects. A classification of 5 types of multimorbidity formation was proposed.

In the discussion on multimorbidity, the important aspect of harmful behavior (lack of exercise, dietary habits, smoking and high alcohol consumption) was hardly taken into account but the results of the follow-up studies (Table 1) underline their impact. Given the importance of these behaviors for the development of multimorbidity and the enormous costs involved, preventive measures to reduce their prevalence should be stepped up, as these are ultimately factors which are can be intentionally influenced. In principle, they can be treated by means of instructions for behavioral modification, i.e. smoking cessation. The difficulty, however, is that these are favored habits with sometimes signs of addiction, i.e. smoking, alcohol consumption. Interventions to change behavior are limited by a low level of education and a low socioeconomic status. Both are also a risk factor for the development of multimorbidity [14, 18, 30]. Therefore, the conditions for successful prevention of multimorbidity are not favorable.

## Limitations

The search strategy has been limited to PubMed possibly missing a significant volume of literature. Furthermore, the methodology and the study design in the cited publications are partly variable and therefore not comparable. More importantly, different definitions of multimorbidity were used. Therefore, these considerations can only be a first attempt to address the complex issue of the development of multimorbidity and its treatment options.
